# Evolution-Based Drug Discovery: Antifungal Also Disrupts Blood Vessel Formation

**DOI:** 10.1371/journal.pbio.1001380

**Published:** 2012-08-21

**Authors:** Robin Mejia

**Affiliations:** Freelance Science Writer, Albany, California, United States of America

**Figure:**
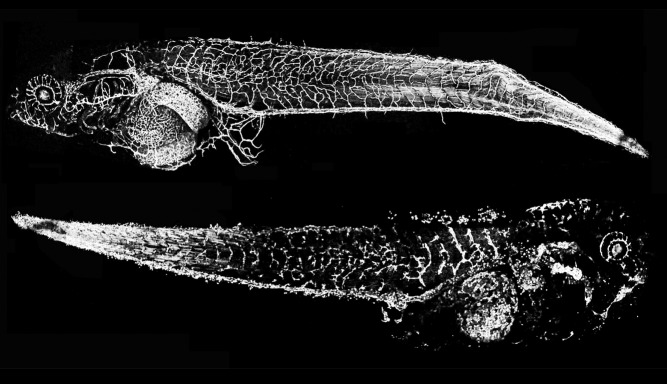
Assaying blood vessels fluorescing in transgenic frogs, thiabendazole, a common antifungal medication, disrupts vessels in the bottom tadpole compared to control treatments to the top tadpole.

Mice, frogs, and even yeast are useful in research in large part because they share so many of our genes. And as a surge of genetic data has become available in recent years, scientists have realized that not just individual genes but entire genetic pathways are often conserved across disparate organisms. In some cases, a genetic module doesn't serve an identical purpose across species; rather, evolution at times repurposes a genetic network. For example, a genetic pathway involved in forming new blood vessels in humans and other mammals is also found in yeast—single-celled organisms with no blood.

To Hye Ji Cha, a graduate student in the laboratories of Edward Marcotte and John Wallingford, the existence of such conserved gene networks suggested a new avenue for drug discovery. Traditionally, scientists identify a single target protein and then screen potential drugs against that target gene. This screening step is often performed in organisms like yeast, and relies on gene-by-gene similarities. Cha and colleagues thought that perhaps researchers could use phenologs—deeply homologous networks of genes that produce different phenotypes when altered—to enable screening against multiple targets in parallel.

In a recent paper, the team described a method for systematically discovering phenologs. Using this approach, they identified the genetic module in yeast that is homologous to one known to promote the development of blood vessels (angiogenesis) in humans. This is exciting because the development of new networks of blood vessels is key to tumor development. Scientists have hoped that drugs that limit blood vessel development could prove useful adjuncts to other chemotherapies. Some such agents are now in clinical trials; however, none have yet been approved for human use.

In this issue of *PLoS Biology*, the researchers take advantage of this newly described genetic homology to demonstrate an evolutionary approach to drug discovery. The results are striking: they show that a drug that's been used as an antifungal agent for 40 years is also a vascular disrupting agent in human cells. At doses analogous to those approved for human use by the United States Food and Drug Administration (FDA), this drug disrupts tumor development in mouse xenografts of human cancer.

After identifying the genetic pathway, the researchers computationally mined an existing chemical sensitivity dataset looking for compounds that interacted with the yeast genes of interest. They then used clustering algorithms to identify candidates based on how they interacted with the yeast genes.

One chemical on the list identified by this search stood out: thiabendazole. Already FDA approved for oral use in humans as an anti-fungal agent, the drug was initially marketed as Montezol from Merck and is now off-patent under several brand names (it is used to control parasites such as roundworm in livestock and on fruits and vegetables to control mold and blight).

The researchers administered the drug to frog embryos, which also have the conserved genetic module, showing that thiabendazole inhibited blood vessel formation. They observed similar results in cultured human endothelial cells. In the endothelial cells, the effect was clearly dose-dependent. They then showed that the drug blocked the effect of vascular endothelial growth factor, a key signal protein required for blood vessel formation.

They tested other commercially available benzimidazoles, but none had a similar effect. (Other drugs in the class caused various developmental defects, but blood vessel formation was not inhibited.)

Notably, the doses of thiabendazole that inhibited angiogensis were in line with the maximum safe dosage for humans.

Next, the researchers sought to understand the drug's mechanism of action. Using time-lapse imaging, they demonstrated that thiabendazole not only prevents new blood vessel from forming, but it also breaks down existing blood vessels. Exposure to the drug caused endothelial cells to round up and retract from each other, and the authors show that the drug disrupts microtubule function, probably by activating a signaling protein called Rho.

Given that thiabendazole is already approved for human use, the results were tantalizing. To get a step closer to real disease, the researchers used a mouse xenograft model of human fibrosarcoma (a cancer of the bones or soft tissue grafted onto a mouse). At doses concordant with the FDA-approved maximum daily dose for humans, the drug slowed the growth of the tumor and led to a smaller final tumor size. As expected, the drug appeared to act by limiting the tumor's blood vessel network—it did not appear to impact the rate of proliferation of tumor cells directly.

The researchers stress that the results are promising on two fronts. Several vascular disrupting agents are in clinical trials, but none have yet been approved for therapeutic use, so the identification of an already approved drug for further study is important.

Additionally, this discovery highlights the value of a new approach to drug discovery. While the techniques of systems biology have led to improved understanding of human diseases and shown promise in identifying disease targets, drug discovery itself has continued to rely on traditional, established methods. This study shows that researchers can take advantage of the genetic homologies supplied by evolution to screen entire genetic modules rather than relying on a traditional gene-by-gene approach.

**Cha HJ, Byrom M, Mead PE, Ellington AD, Wallingford JB, et al. (2012) Evolutionarily Repurposed Networks Reveal the Well-Known Antifungal Drug Thiabendazole to Be a Novel Vascular Disrupting Agent. doi:10.1371/journal.pbio.1001379**

